# The absorption of ultrasound in emulsions: computational modelling of thermal effects

**DOI:** 10.1038/s41598-018-30664-7

**Published:** 2018-08-21

**Authors:** Derek Michael Forrester, Valerie J. Pinfield

**Affiliations:** Loughborough University, Chemical Engineering, Loughborough, LE11 3TU United Kingdom

## Abstract

Around liquid particles in a fluid of contrasting properties (for example, oil in water) in ultrasonic fields there are small regions where thermal waves can propagate with relatively high amplitudes. Herein, we demonstrate the existence and character of these waveforms using three-dimensional finite element modelling based on linearised Navier-Stokes equations. We investigate single particles and small clusters of particles, validating the expected thermal wavelength and the power dissipation due to viscous and thermal effects around the particle. The energy lost due to thermal and viscous dissipation is explored as a function of the average separation distance between the particles (linking to concentration) as well as the applied frequency. The determination of energy loss provides a new method for calculating the attenuation in particle systems. We demonstrate that the effective attenuation of an emulsion in which particles exist in clusters is influenced by the interparticle separation within the cluster, even at the same total particle concentration. Thus, the finite element modelling provides evidence for thermal interactions and their effect in correlated particle systems.

## Introduction

Flow dynamics inclusive of internal friction were described by Navier^1^ and others. Notably, Stokes also added viscous effects to the equations of motion and investigated their influence on sound propagation in a medium^2^. Kirchoff later studied sound attenuation and heat conduction^3^. Thus, there were discovered two forms of classical sound attenuation mechanisms in homogeneous media - those associated with viscous- and thermal-related losses. Indeed, the temperature change induced by absorption of intense, focussed acoustic fields is exploited in medical therapeutics; the thermal fields can be visualised with Magnetic Resonance Imaging and other imaging techniques^4,5^. In inhomogeneous materials, such as particles in a fluid, additional viscous and thermal effects occur near interfaces. Meyer and Güth, for example, performed experiments in 1953 using charged oil drops that traced the viscous boundary layer in a sound wave^6^.

The topic of this paper concerns linear ultrasound fields of low amplitude, where temperature perturbations in the bulk liquids due to the compressional perturbations of the acoustic wave are small (of order milli-Kelvin). These are associated with absorption by thermal dissipation which (although the temperature amplitude is small) causes a detectable contribution to the attenuation of the acoustic wave in a homogeneous liquid, such as water or oil. Here we address the additional attenuation of a linear acoustic wave caused by the presence of particles of contrasting physical properties (compressibility, density and thermal properties) which gives rise to thermal waves of small temperature amplitude (milli-Kelvin) emanating from the particle surface and dissipating energy locally to that surface. These thermal waves are induced by the difference in the temperature perturbation produced in the particle and in the surrounding liquid by the compression caused by the acoustic wave. This mismatch produces an oscillatory temperature-dominated compressional wave (referred to as a thermal wave) which decays rapidly with distance from the particle surface, dissipating its energy in that region. The thermo-acoustic scattering phenomenon leads to an increase in the attenuation of a linear acoustic wave propagating through suspensions of particles in a liquid in the long acoustic wavelength regime, as demonstrated experimentally by McClements and Povey amongst others^7^. A corresponding effect arises due to density contrast, leading to short-range shear waves emanating from the particle surface and dissipating energy by a viscous dissipation mechanism in the region near the particle surface. Such visco-acoustic effects are predominantly observed in the increased attenuation of low power acoustics fields in solid particle in liquid suspensions and are typically small in oil in water systems.

In some industrial processes, suspended particles in liquids (such as oil droplets in water) are subjected to an applied sound wave, for the purpose of process monitoring and particle characterisation, a technique known as ultrasonic spectroscopy^8,9^. The acoustic fields are of low intensity (linear), and measurements of attenuation (or other acoustic characteristics) are interpreted in terms of models of acoustic propagation in the inhomogeneous medium. The short-range thermal and/or viscous fields near the particle interfaces^10^ dissipate energy into the suspending medium usually without strongly changing the particle coupling^11^, for highly diluted systems and low power ultrasound. The contribution of these effects to attenuation in inhomogeneous media has been known for some time and can be defined using a model for the scattering of an acoustic wave by a single particle^12^, together with a model for the combined effects of many particles, either assuming independence of the particles (following Epstein and Carhart^12^), or accounting for the multiple interactions of the acoustic fields with particles through a multiple scattering model, reviewed by Challis *et al*.^8^. However, as the concentration increases, the separation distances between particles decreases and the decay fields that are produced can interact; that interaction can cause a significant change in the attenuation of the acoustic wave^10,13^. Such interaction of the thermal and shear fields between particles is not accounted for in the acoustic scattering models, and recent attempts to develop alternative models have been only partially successful when compared with experimental data^14–16^. For oil-in-water emulsions studied in this work, the visco-acoustic scattering is expected to be relatively low, due to the similarities of the densities of typical oils and water. However, relatively large thermo-acoustic effects arise in the oil-in-water suspensions with radial, pulsatory thermal wave fluctuations from the oil/water boundaries due to the contrast in thermal and physical properties of the oil and water. In sum, these contrasting properties, the size of the oil particles, the incident wave frequency and the particle concentration control the thermal losses in the suspension. Here we investigate through finite element modelling these thermal effects arising near particle boundaries and investigate the effect of particle separation on attenuation losses.

The energy losses for a propagating, linear, planar, acoustic wave through a suspension of oil particles in a liquid is defined by an attenuation coefficient, *α*, such that

1 $$E={E}_{0}{e}^{-2\alpha z}$$

where the energy flux in the suspension at position *z* is *E* and the energy flux in the suspension at the position *z* = 0 is *E*_0_. The attenuation coefficient has contributions due to bulk thermal and viscous losses in the surrounding water phase, acoustic scattering (re-direction) of the incident plane wave by the particle, viscous and thermal losses due to dissipation in the small boundary layers near the particle surface, and the effects of interaction of thermal and shear waves between particles^14,16,17^.

We are interested in determining the thermal and viscous fields around a single scattering particle of oil to find the energy dissipation per unit time. This power dissipation is discussed in the classical work by Epstein and Carhart (EC) as the dissipation function (a combination of the thermal and viscous dissipation functions)^12^. The EC analysis from 1953 makes a number of assumptions for the analysis of systems of many particles of the liquid-in-liquid type, but an important one is that the total energy loss in the system can be found by a multiplication of the energy loss caused by a lone scatterer by the number of particles in the system^12^. This assumption is fine for dilute systems because the particles will be separated by distances greater than at least several thermal wavelengths so that the separation is larger than the thickness of the thermal boundary layer around each particle. However, this leaves open the question of what influences neighbouring particles will have on the overall attenuation when concentrations are higher and the gap between particles is of the same order or smaller than the thermal boundary layer, such as in aggregated systems^18^. As concentration increases, thermal and shear effects (predominantly in solid particle in liquid systems) in an ultrasonic field can be strong enough to produce a significant reduction in attenuation as a result of the interactions of the thermal and shear fields of neighbouring particles^10,13–16^. Modifications to the multiple-scattering models to account for these effects have found partial success^10,13,14,16^ but there is a need for greater understanding of the effects of interactions at the particle level, and for a means to verify or test proposed models numerically alongside experimental measurements where other uncertainties (e.g. particle size distribution, physical properties, dispersion etc.) can cause difficulty.

Herein, we choose to investigate by finite element modelling, particle/ultrasonic wave interactions in the regimes where the thermal wave numbers multiplied by the particle radii give dimensionless numbers close to one, i.e. *k*_*T*_*r* ≈ 1 where thermal effects are strongest^19^. We consider spherical microparticles of 0.25 *μ*m diameter composed of sunflower oil in water, in the frequency range 1–10 MHz. For a single particle and for small clusters, three-dimensional modelling of the particles as spheres is conducted using Comsol Multiphysics version 5.3 and the Thermoviscous Acoustics package. In a recent paper, we have thoroughly validated a 2D axisymmetric finite element model using the Thermoviscous Acoustics package for a single oil particle and a single solid particle in water by comparison with the analytical model of Epstein and Carhart^12,20^. Here we extend the investigation to the power dissipation due to the thermal and viscous fields around a single particle, and the interactions between a small cluster of closely-spaced particles using a 3*D* model. Due to the high computational demands associated with 3*D* simulations we are restricted to studying interacting particle problems in the space occupied by particles within a small number of thermal wavelengths. However, this will prove to be sufficient to demonstrate confinement effects related to the proximity of the particles and to show that theory and numerical modelling of the thermal decay field agree. Thus, we will begin by analysing single particles of sunflower oil in water of 0.25 *μ*m diameter and their thermal fields, focussing on the energy dissipation in the region of the particle. Then we demonstrate the behaviour of the decay fields in small clusters of seven particles where particles are clustered more or less loosely; this technique is then used to predict the attenuation in emulsions. Simulations were conducted for a frequency range of 1–10 MHz with an incident planar pressure wave of 0.1 MPa pressure amplitude, using linear acoustics.

## Results

### Single oil particle analysis

The ultrasonic pressure wave fluctuations around the scatterer produce oscillations in temperature in the vicinity of the oil/water interface. Since the oil and water have different physical properties, the same compression produces a different temperature change in the two materials. Thus an additional wave is excited due to the continuity condition on temperature at the surface; this wave is the thermal wave which is associated with relatively large temperature changes and small compression changes compared with an acoustic wave. The heating and cooling near the boundary is associated with the pulsation of the oil droplet that creates a secondary sound source as well as the monopolar thermal wave. Using finite element modelling the energy loss mechanisms of the oil-in-water system can be elucidated. First it is necessary to demonstrate that the FEM is capable of producing accurate thermal wavelengths for the thermal wave as compared to the predicted values given by,

2 $${\lambda }_{T}=2\pi \sqrt{\frac{\kappa }{\pi \rho f{C}_{p}}}=2\pi {\delta }_{T},$$

with *κ* and *C*_*p*_ the thermal conductivity and specific heat capacity of the suspending medium, respectively. Coexisting with the thermal boundary layer is a viscous boundary layer. Suitably far from the oil particle the compressions and expansions of the applied field occur adiabatically. Near the particle boundaries there are localised isothermal effects. The visco-thermal relationship for the boundary layers is given by the Prandtl number, which is commonly used in the description of heat transfer in fluids,

3 $${P}_{r}=\frac{\mu {C}_{p}}{\kappa }$$

Thus,

4 $${\delta }_{visc}=\sqrt{\frac{\mu }{\pi \rho f}}=\sqrt{\frac{\kappa {P}_{r}}{\pi \rho f{C}_{p}}}=\sqrt{{P}_{r}}{\delta }_{T}$$

**Figure 1:**
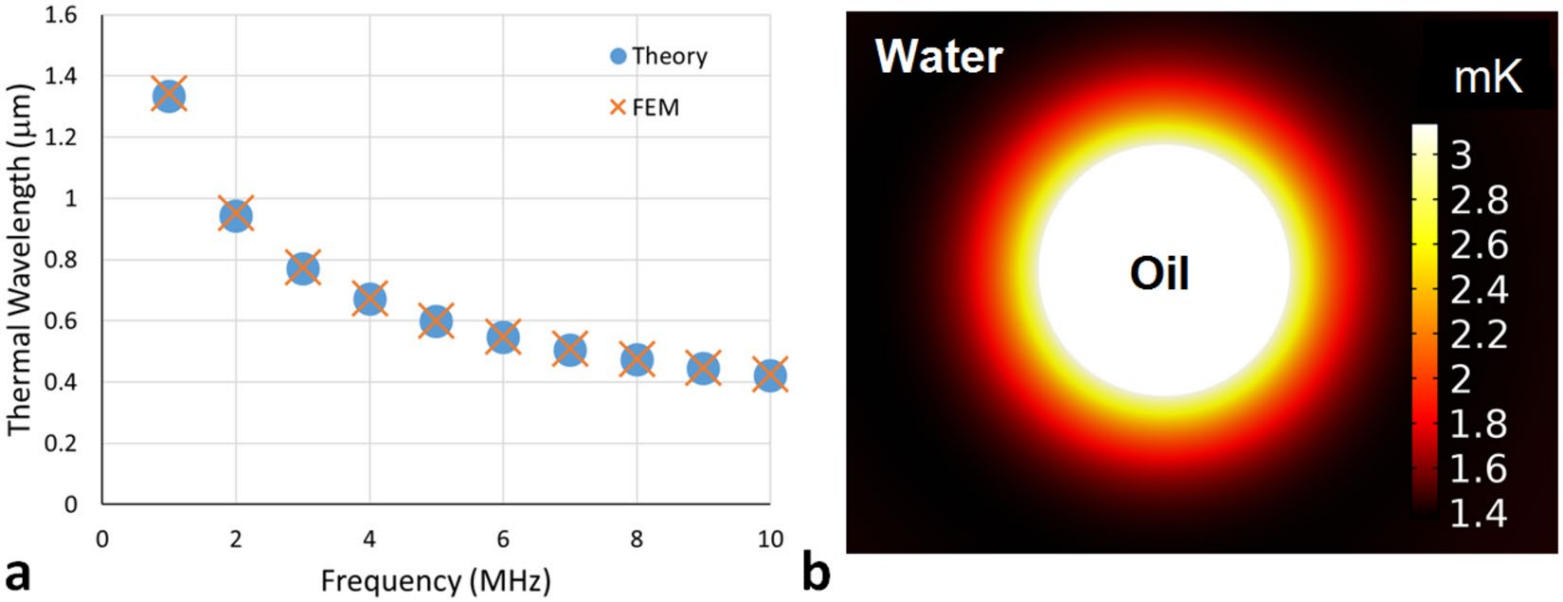
The thermal wavelength calculated using finite element modelling (FEM) and equation (2). In the FEM result a maximum element size of *λ*_*T*_/20 is used. (**a**) Comparison of theory and FEM, with deviation from theory of several nanometres. (**b**) The thermal wave is monopolar around the particle as shown here at 8 MHz, where the temperature perturbation is plotted through the use of the colour bar on the right, showing its spherically symmetric nature.

Figure 1 demonstrates the comparison of the FEM thermal wavelength against that obtained from Eq. (2). The thermal wavelengths are found using FEM by finding the intervals between successive maximina and minima of the temperature perturbation, which relates to the existence of the thermal wave. The intervals are expected to be equivalent to half the thermal wavelength, therefore enabling the wavelength to be determined. The values determined from the finite element model match the theoretical values very well in the frequency range 1–10 MHz investigated here, with deviations from the theoretical value of only a few nanometres. The thermal wavelengths are of order micrometers in this frequency range, and since the thermal wavenumber has equal real and imaginary parts, the decay of the wave occurs over a similar length scale. Figure 1 also illustrates the temperature perturbation field around the particle, demonstrating its monopolar nature.

**Figure 2:**
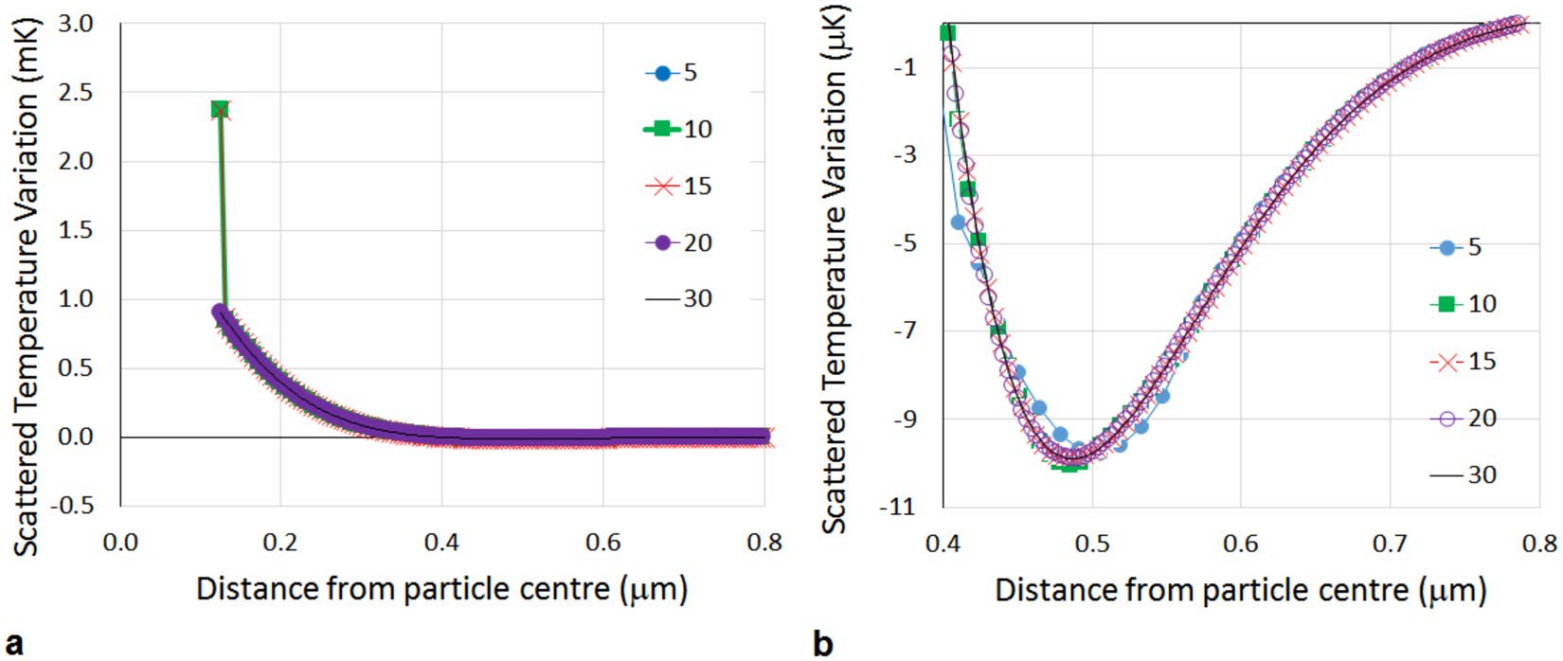
The temperature perturbation of the scattered field as a function of distance from the particle centre for a single particle at a frequency of 3 MHz. The legend shows the minimum number of mesh elements per thermal wavelength (*n*) such that the maximum mesh element size was *λ*_*T*_/*n*. (**b**) A magnification of (**a**) where the curve of the temperature perturbation appears to have plateaued. In (**b**), in the region 0.4–0.8 *μ*m from the particle edge, the first minimum of the thermal wave can be seen.

**Figure 3:**
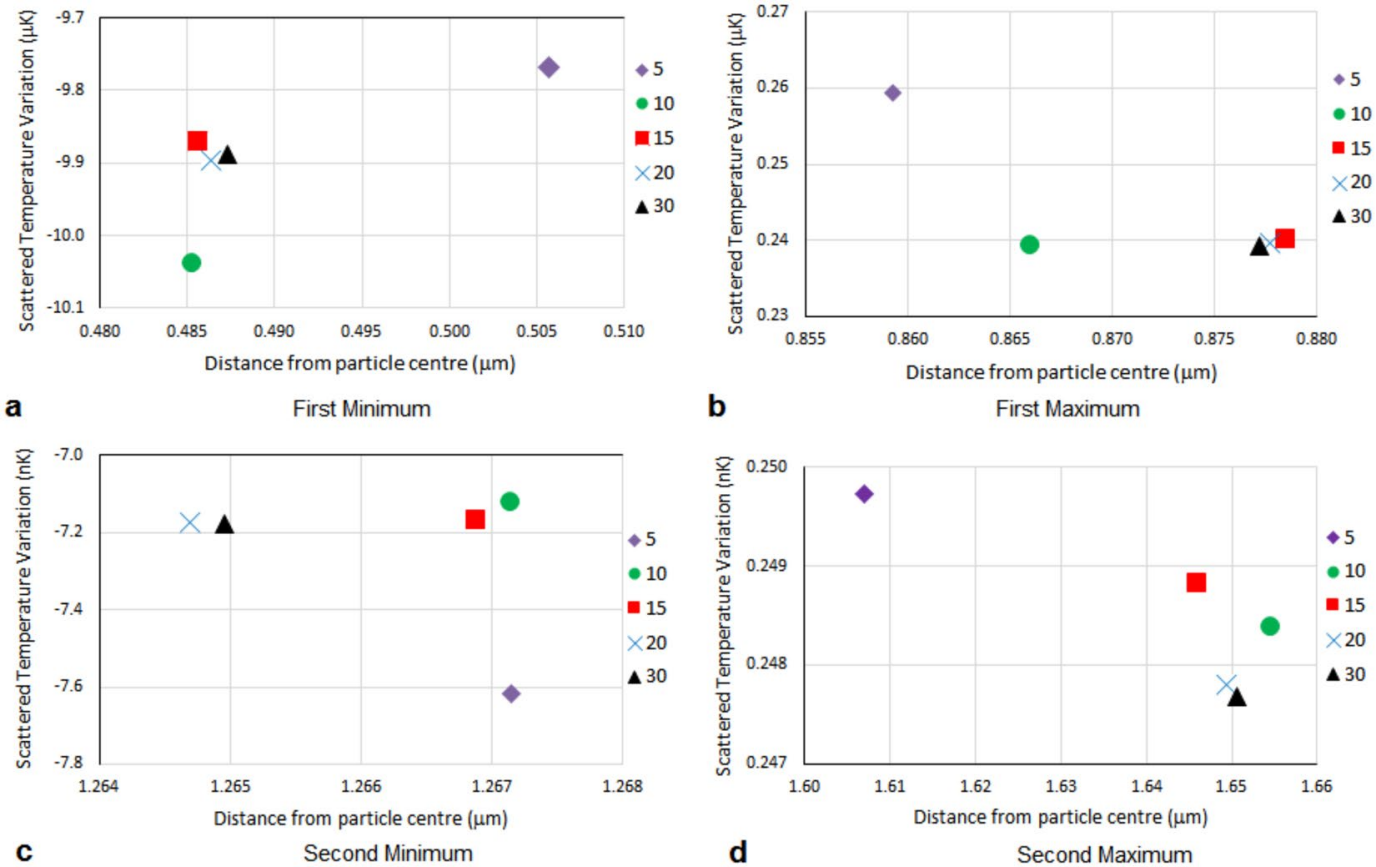
The locations and temperature magnitudes at the turning points (maxima and minima) in the temperature perturbation over two wavelengths 2*λ*_*T*_, showing the effect of mesh refinement for a single particle at a frequency of 3 MHz. The legend shows the minimum number of mesh elements per thermal wavelength (*n*) such that the maximum mesh element size was *λ*_*T*_/*n*.

The FEM analysis is conducted with a maximum element size imposed of *λ*_*T*_/20; the validity of this limitation is demonstrated in Figs 2 and 3 showing results for different choices of maximum element size, ranging from *λ*_*T*_/5 to *λ*_*T*_/30. Figure 2 shows the temperature perturbation of the scattered field as a function of distance from the particle centre, demonstrating that the temperature at the surface is of order milli-Kelvin, but since the thermal wave is exponentially decaying over a micrometre length scale, wave-like oscillations are difficult to identify on a single graphical scale (Fig. 2(a)) and are shown on an expanded scale in Fig. 2(b) to highlight the first minimum. The results show that the temperature perturbation at the surface does not converge as the element size is decreased until a maximum element size of *λ*_*T*_/20. Maximum element sizes corresponding to both 10 and 15 elements per thermal wavelength predict significantly different values for the surface temperature. Although this temperature perturbation is small, it is known through experimental evidence that such effects have a significant impact on measured attenuation^10,16^. Figure 3 presents the temperature variation and locations of successive maxima and minima. It can be seen that although improving the mesh refinement from the coarsest mesh modifies the amplitude and locations of the turning points, the results for *λ*_*T*_/20 and *λ*_*T*_/30 are very similar and so it makes computational sense to use the lower limit of these converged solutions i.e.*λ*_*T*_/20. A balance between maximum element size, resolution of the rapidly decaying wave amplitude, and computation efficiency is met.

Having established that the FEM is consistent with the expected thermal wavelength, the energy loss for a single particle is now investigated. The time-averaged power (energy per unit time) in an incident wave passing through a unit cross-sectional area (i.e. normal to the propagation direction) is,

5 $${P}_{0,A}=\frac{1}{2}k\omega \rho {|{{\rm{\Phi }}}_{0}|}^{2}=\frac{{p}_{0}^{2}}{2c\rho }$$

where the amplitude factor from the incident velocity potential,

6 $${{\rm{\Phi }}}_{0}=\frac{i{p}_{0}}{\omega \rho }.$$

**Figure 4:**
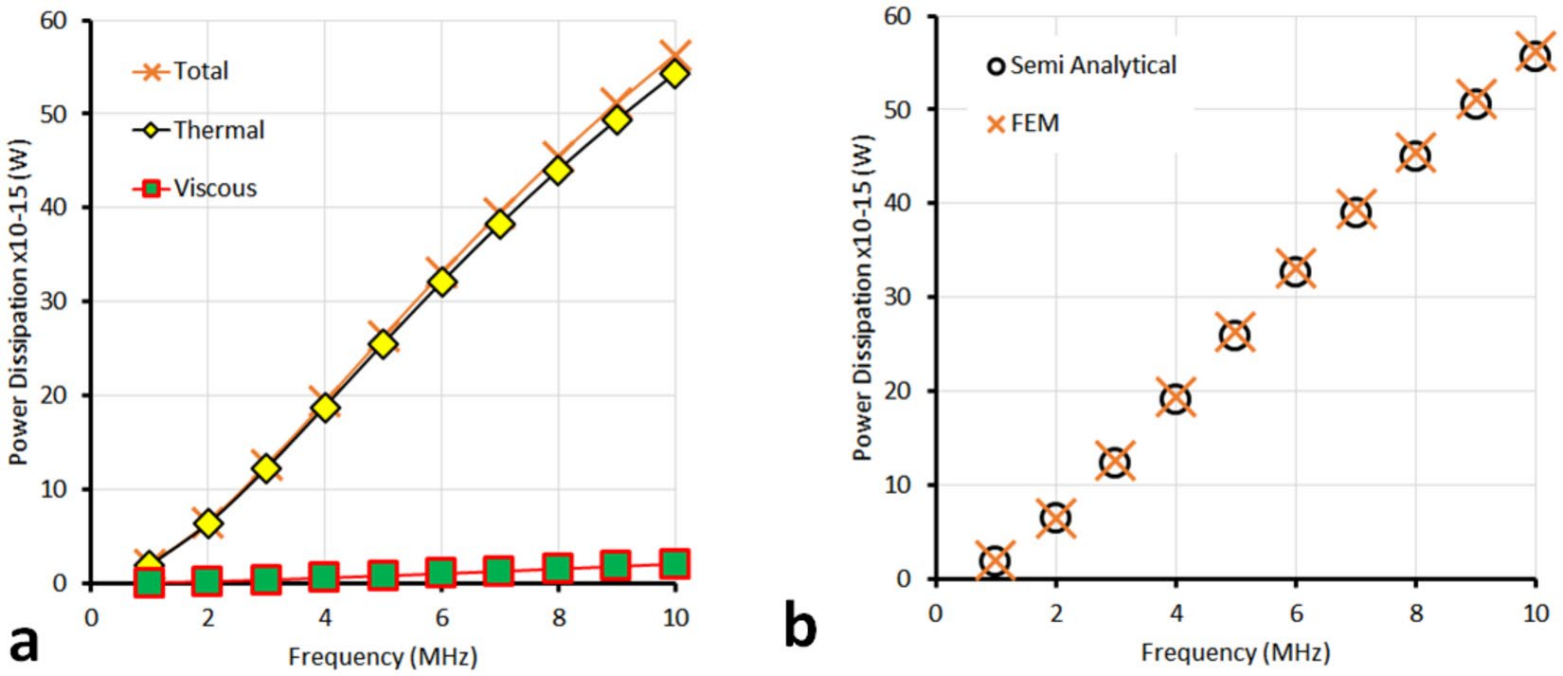
(**a**) The scattered power dissipation, ${\tilde{P}}_{sc,1}$, for a single sunflower oil particle of diameter 0.25 *μ*m in water (orange crosses), determined by the FEM method using Eq. (10), is the sum of the viscous and thermal dissipation densities (green squares and yellow diamonds, respectively) with the bulk water values subtracted in each case. (**b**) Comparison of the semi-analytical result (Eq. (9)) for determining the scattered power dissipation with that found using FEM.

Equation (5) is similar to that found in Epstein and Carhart^12^, section 14, but with the inclusion of (6) giving the incident wave amplitude. Here, the tiny attenuation loss due to the shift of the origin (from the centre of the particle in EC to the edge of the domain in the FE model) is neglected. The dissipated power consists of two terms: that due to the bulk properties of water and that due to the scattering. The time averaged power per unit volume dissipated in the bulk liquid is given by viscous and thermal contributions such that

7 $${P}_{bulk,visc}=\frac{{\omega }^{2}}{\mathrm{2(}\rho {c}^{2}{)}^{2}}{p}_{0}^{2}\mu (\frac{4}{3}+\frac{{\mu }_{B}}{\mu })$$

and

8 $${P}_{bulk,therm}=\frac{{\omega }^{2}}{2{(\rho {c}^{2})}^{2}}{p}_{0}^{2}\frac{\kappa (\gamma -1)}{{C}_{p}},$$

where *μ*_*B*_ is the bulk viscosity, *μ* is the shear viscosity, and *κ* is the thermal conductivity. For a single particle, the time-averaged power dissipation due to scattering (sc) is

9 $${P}_{sc,1}=W{|{{\rm{\Phi }}}_{0}|}^{2}=-\,2\pi \rho \frac{{p}_{0}^{2}}{\omega k\rho }\Re ({A}_{0}+3{A}_{1}),$$

where *W* is the power dissipation given by Epstein and Carhart in their equation (14.1), and *A*_0_ and *A*_1_ are the zero-order and first order scattering coefficients, respectively^12^. The losses are due to dissipation in the thermal and shear waves very close to the particle boundary. To obtain the total power dissipation, the bulk dissipation in water must be considered in addition to the scattered power loss. To compare the analytical result for the scattered contribution to power dissipation with those from FEM, the power dissipation from scattering must be calculated from the total power dissipation in a volume enclosing the particle, minus the power dissipation in that volume as if it were pure water (i.e. the suspending medium):

10 $${\tilde{P}}_{sc,1}={\tilde{P}}_{tot}-{\tilde{P}}_{bulk},$$

where the tilde represents the power dissipation densities calculated in FEM. Figure 4 compares the scattered power dissipation found analytically by Eq. (9) with that of the FEM analysis using Eq. (10) for a single particle at a range of frequency from 1–10 MHz. The FEM result ${\tilde{P}}_{sc,1}$ is found through the total power dissipation with and without the scatterer (with the scatterer replaced by water using the same mesh) as prescribed using a combination of the thermal and viscous components of the built-in power dissipation function in Comsol. The bulk dissipation power can also be calculated analytically (see for example Pierce^21^, and see Table 1). The values of the data points in Fig. 4 can be seen in Table 1. The plot of power dissipation versus frequency shows that in this case of an oil particle in water, the thermal losses due to scattering dominate the shear losses due to scattering. The results for power dissipation obtained by the FEM method and the semi-analytical method agree very well for the single oil particle in water case. Thus, the FEM model predicts accurately the energy losses in the case of the single particle. Having demonstrated this, we now look at the energy losses for small clusters of particles, in which the effects of thermal interactions between particles can be explored.

**Table 1:** Comparison of the values of the bulk (water) and scattered (oil in water) dissipation powers (*P*) found from the FEM modelling with the method described by Eq. (10) and the analytical formula of Eq. (9). Columns with a power marked with a tilde are results from the FEM.

Frequency (MHz)	${\tilde{{\boldsymbol{P}}}}_{{\boldsymbol{bulk}}}$	*P*_*bulk*_	${\tilde{{\boldsymbol{P}}}}_{{\boldsymbol{sc}}{\boldsymbol{,}}1}$	*P*_*sc*,1_(×10^−15^W)
1	1.55	1.67	1.93	1.89
2	6.18	6.66	6.50	6.39
3	13.91	14.99	12.57	12.37
4	24.72	26.64	19.34	19.06
5	38.63	41.63	26.27	25.93
6	55.62	59.94	33.05	32.64
7	75.71	81.59	39.49	39.02
8	99.89	106.56	45.51	44.99
9	125.15	134.87	51.11	50.54
10	154.50	166.51	56.30	55.68

### Small cluster analysis

A small cluster of 7 equally spaced particles was modelled using FEM, with a central particle surrounded by 6 others located on the Cartesian axis directions. If each particle behaved independently in the acoustic field, the total power dissipation (due to scattering, excluding the bulk dissipation) would be expected to be a multiple of 7 times the power dissipation for a single particle (obtained in the previous section). Epstein and Carhart made this assumption for ensembles of particles, thus deriving an estimate for the attenuation losses for an acoustic wave propagating through such an ensemble. The FE model of the cluster was used to investigate the effect of the distance between the particles on the total power dissipation in the system using separations between the particle surfaces of 0.05 − 0.25*λ*_*T*_ at a frequency of 3 MHz. The integrating volume for the power dissipation was defined as a sphere of radius 0.875 *μ*m concentric with the central particle.

**Figure 5:**
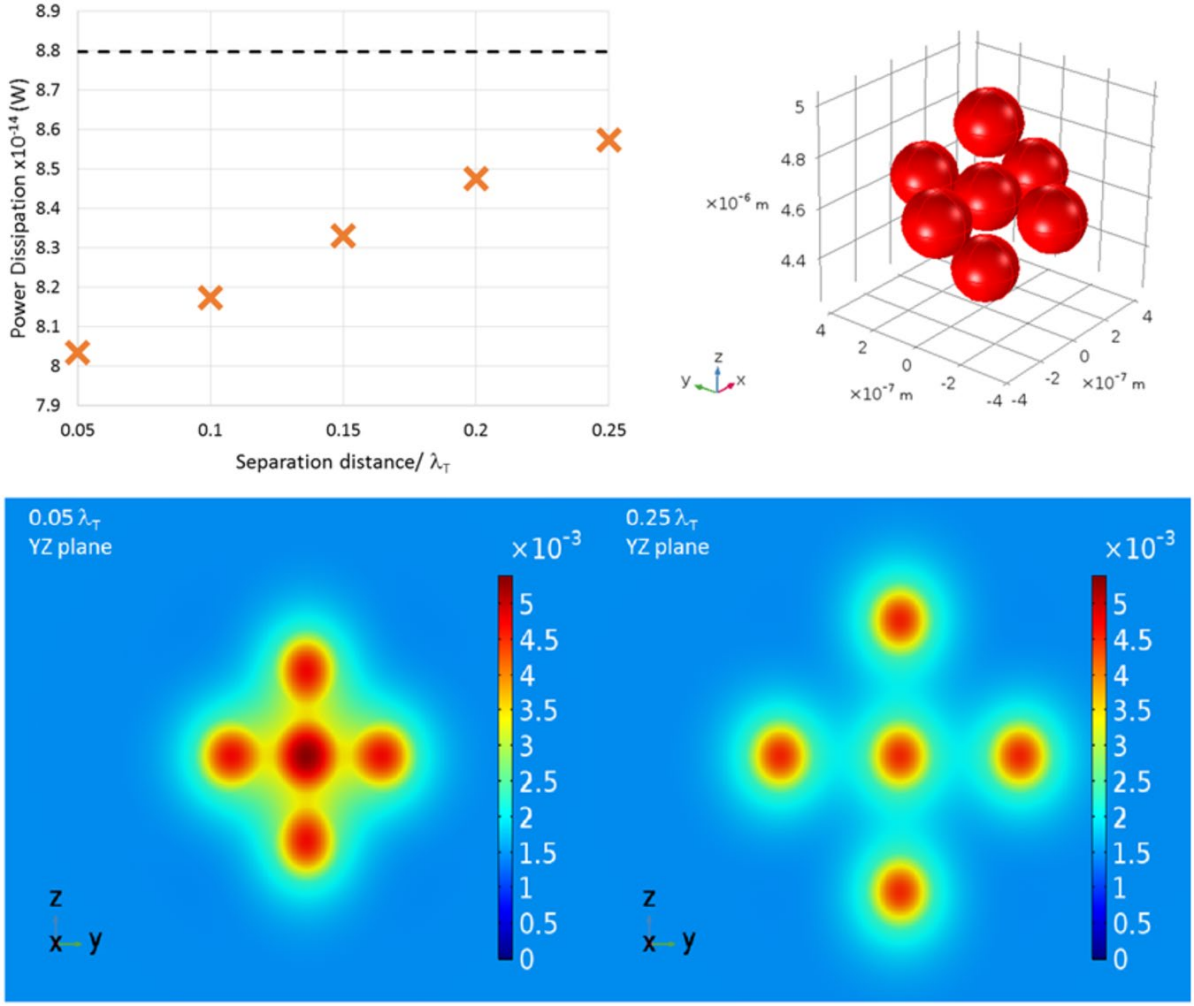
Top Left: The total power dissipation at 3 MHz in a volume with radius 0.875 *μ*m including seven particles, all with equal separation, minus the power dissipation in that volume as if it were pure water. The separation distance varies from 0.05*λ*_*T*_ to 0.25*λ*_*T*_. The dashed line represents the dissipation calculated through the use of the single particle approximation, obtained through FEM, i.e. $7\times {\tilde{P}}_{sc,1}$. Top Right: The geometric arrangement of the system of seven oil particles. Bottom: The thermal fields around the central five particles are shown in the *y* − *z* plane slicing through the particle centres for separation distances of 0.05*λ*_*T*_ and 0.25*λ*_*T*_ (units in Kelvin).

Figure 5 shows the scattered power dissipation plotted as a function of the particle separation, compared with the result expected for non-interacting particles (=7× the single particle result). As the separation increases the power dissipation increases towards the level for the non-interacting particles. However, the scattered power dissipation is reduced by around 10% for particle separations of 0.05*λ*_*T*_ and 5% for separations of 0.15*λ*_*T*_. The reduction in power dissipation is due to the interaction of the thermal fields around the particles, as illustrated by the temperature field maps in the y-z plane shown in Fig. 5. At small interparticle separations, an elongated field pattern is observed, with deviation from sphericity. As particles move closer together, the combined thermal fields around the particles results in a lower thermal dissipation than if those particles were far apart and the thermal waves dissipated completely in the region of the particle. Here, at close interparticle separations, some energy is retained in the acoustic field by the interaction of the thermal fields of neighbouring particles.

### Attenuation

Having considered the scattered power dissipation for a small cluster of particles, and the effect of the interparticle distance on that scattered power, the link with ensembles of particles in a suspension or emulsion is now considered. The power dissipation is closely related to the effective attenuation of an acoustic wave in the medium, and the effect of interparticle separation on power dissipation will be observed in effective attenuation. The contribution to the amplitude attenuation (half the energy attenuation) due to scattering is related to the scattered power dissipation per unit volume (*P*_*sc*,*V*_) by

11 $${\alpha }_{sc}=\frac{{P}_{sc,V}}{2{P}_{0,A}}$$

and the total amplitude attenuation is obtained by combining with the attenuation in bulk water

12 $$\alpha ={\alpha }_{sc}+{\alpha }_{w}$$

Considering an ensemble of clusters of the type considered in the previous section, the scattered power dissipation per unit volume for the ensemble is given by the number of clusters per unit volume multiplied by the scattered power dissipation for each cluster, assuming that each cluster behaves independently. Thus

13 $${P}_{sc,cl,V}={n}_{cl}{P}_{sc,7,d}$$

where *P*_*sc*,*cl*,*V*_ is the scattered power dissipation per unit volume in the ensemble of clusters, *n*_*cl*_ is the number of clusters per unit volume and *P*_*sc*,7,*d*_ is the scattered power dissipation for a single cluster containing 7 particles at the interparticle separation *d*, which is plotted in Fig. 5. Thus the effective attenuation through an emulsion comprising of clusters of 7 particles with various separations can be determined.

The clusters were assumed to be spherical with a radius of 7 times the particle radius (i.e. 0.875 *μ*m); this is the domain size in the finite element model, and at the largest interparticle separation (0.25*λ*_*T*_) this allows the “surface” of the cluster to be around 0.4*λ*_*T*_ away from the outermost particles. The cluster size permits substantial decay of the thermal wave outside the outermost particles so that clusters act independently, but some small interactions may remain in the system of an ensemble of clusters which are not accounted for here. Clusters are assumed to have no interaction and to act independently. At a concentration of clusters of 49v/v% the total volume fraction of particles in each system is 1v/v%.

For comparison, the attenuation expected for a randomly dispersed emulsion where each particle acts independently is also determined. This is calculated using the formula

14 $${P}_{sc,V}={n}_{p}{P}_{sc,1}$$

where *P*_*sc*,*V*_ is the effective scattered power dissipation per unit volume in the system, *n*_*p*_ is the number of single particles per unit volume and *P*_*sc*,1_ is the scattered power dissipation for a single particle. This is combined with the attenuation formulae given above to obtain the attenuation in the system.

**Figure 6:**
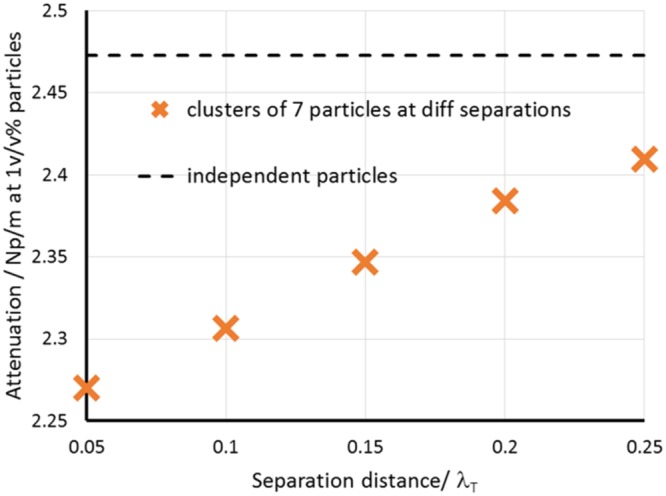
The attenuation for clusters of seven particles at 1v/v% with different separations compared to that found for independent particles.

Figure 6 shows the calculated results for effective attenuation for the system of clusters with varying interparticle separations within the clusters, and for a system of independent particles at the same concentration. It should be noted that in every case, the volume fraction of particles in the emulsion is 1v/v%. The dashed line shows the expected attenuation if each particle acted independently in an acoustic sense, with no interaction between the thermal fields (or indeed acoustic fields) of each particle. However, the results for the attenuation for the ensemble of clusters indicate that the attenuation can be markedly reduced (even at the same overall concentration of particles) when the particle locations are correlated such that the particles are grouped in clusters. As the particle separation within the clusters is increased, the attenuation tends towards the limit of independent particles. However, the restrictions on computational resources limited the particle separation that could be modelled and the cluster did not reach the limit of independence of the thermal fields within the cluster. At small interparticle separations, the attenuation is significantly lower than in the independent particle case.

## Discussion

The validity of the single particle approximation for emulsions of low concentration has been verified herein. However, when the concentration of oil increases and an applied ultrasonic wave propagates through the system, local thermal gradients emerge in the vicinity of the oil droplets. These decay exponentially, but at the higher concentrations, coupling between particles due to the thermal waves produces a strong influence upon the attenuation. By modelling the system based on finite element methods we are able to get a deeper insight into the physics occurring within and around particles in an emulsion. As the concentrations increase and the spherical oil particles become more closely packed, making the separation distance a function of the thermal wavelength allows the model to fully incorporate the effects of the overlap fields and to more accurately model attenuation. We have investigated a symmetric system where the degrees of freedom around a single oil particle are covered by six nearest neighbours. By inclusion of small numbers of neighbouring particles the thermal waves interference effects are included in the model. We found that the FEM method is suitable as a means to determine the attenuation for clusters of groups of particles. Although a compute-intensive technique for prediction of attenuation, the model offers the potential for systematic investigation of particle interaction effects which do not yet have a corresponding analytical model to describe them.

We have demonstrated that the attenuation in a system where particles are in small clusters can differ significantly from the attenuation in a system of the same concentration where particles are well-dispersed. For a system of independent scatterers, an attenuation of 2.47 Npm^−1^ was determined by the finite element model for the 1%v/v sunflower oil in water system with particles of diameter 250 nm at 3 MHz. Experimental data for such a system could not be found in the literature, but experimental data for a similar system of 5%v/v corn oil in water of diameter 240 nm have been published by Chanamai *et al*.^10^ over a wide frequency range (their Fig. 1). Scaling their data (assuming linear dependence on concentration at these low concentrations) leads to an experimentally-measured attenuation of 2.0 Npm^−1^ at 1%v/v at 3 MHz. This is in reasonable agreement with our finite element simulation results for independent scatterers, considering the difference in oil properties between the simulated and experimental systems and the polydispersity of the experimental system (the quoted diameter is the *d*_32_ value). Comparison with experimental data for aggregated systems is of limited value, because the cluster system simulated here is artificial due to the constraints of computational resource. The simulations serve to illustrate the potential effect of interparticle separation on attenuation, but the simulated cluster structure and their small nature limit any comparison with experimental data. The results are consistent qualitatively with experimental findings in aggregated or concentrated emulsion systems^10,13,14,16^, in which the attenuation is found to be lower than that predicted for a system of independent scatterers where thermal dissipation around the particles is included but where those thermal fields do not interact. It has been observed experimentally that reductions of up to 50% in attenuation can occur compared with the models neglecting thermal and shear interactions, at particle concentrations of 20%v/v for solid particle^14^ and 50%v/v for liquid particles^10^. Here, at very low simulated concentrations of 1%v/v, a reduction of 10% has been predicted for the highly-structured, low density cluster system. This is within the ranges observed experimentally. Any further quantitative comparison must be reserved until simulations can be constructed for larger ensembles of particles and more realistic cluster structures where particle configurations can be statistically defined to model variations in the distribution of interparticle separations. Similarly, highly controlled and fully-characterised experimental systems are required to validate such models. Such experimental data does yet exist to the authors’ knowledge.

FEM inclusive of thermo-viscous effects is, therefore, a powerful technique for analysing the physical characteristics of an emulsion. It is the influence of the thermal fields on neighbouring particles that can be investigated using the FE model to support the development and validation of new models for attenuation in concentrated and aggregated systems. Such systems are of interest for food, pharma and healthcare products and their characterisation by ultrasound relies on a thorough understanding and physically valid model of the energy dissipation phenomena. Here, particles of equal sizes (250 nm diameter) were investigated, but FEM can easily be used to model a realistic particle size distribution, as obtained experimentally. Equally the methods herein can be adapted for use with solid-in-liquid systems and may be useful where multiple scattering theory breaks down at high concentrations. Random positioning of the particles can also be included in the modelling so that some particles are closer and some further apart, giving flexibility to the approach to enable investigation of the effect of particle position correlations on the attenuation. Non-spherical particles^22^ are also easily adopted into the method. Thus, we have validated the single particle approximation of Epstein and Carhart^12^ for dilute systems and shown that by inclusion of boundary layer effects higher concentration systems can be modelled over a broad bandwidth of frequencies. These techniques open a new avenue in research for use in particle characterisation and novel lossy materials with complex effective properties^23^ and to understand better the short and long range interactions between particles in suspensions.

## Methods

### Material properties

The physical properties of the oil and water used in the model are shown in Table 2. Ultrasonic spectroscopy enables the determination of the attenuation and from a theoretical analysis based on the Navier-Stokes equation a value of the bulk viscosity can be determined. For water, which is a Newtonian fluid, the acoustic attenuation scales as the square of frequency, giving a bulk viscosity that is stable over a large frequency range. However, oils are typically non-Newtonian fluids and the bulk viscosity varies with frequency because of the attenuation scaling as a function of *f* ^*n*^ where $n\ne 2$. The bulk viscosity, *μ*_*B*_ is given as^11,24^,

15 $${\mu }_{B}=\frac{2\alpha \rho {c}^{3}}{{\omega }^{2}}-\,\frac{4\mu }{3}-\,\frac{(\gamma -1)\kappa }{{C}_{p}}$$

**Table 2:** Properties of oil and water at 293015 K.

Property	Water	Sunflower Oil
Speed of sound, *c* (ms^−1^)	1482.3	1469.9
Density, *ρ* (kgm^−3^)	998.2	920.6
Isothermal compressiblity, *β*_*T*_ (GPa^−1^)	0.459	0.584
Shear viscosity, *μ* (mPa.s)	1	54
Thermal conductivity, *κ* (J(msK)^−1^)	0.591	0.17
Specific heat capacity, *C*_*p*_ (J(Kg K)^−1^)	4182	1980
Bulk viscosity, *μ*_*B*_ (mPa.s)	2.45	see Table 3
Ratio of specific heats, *γ*	1.0068	1.1613
Coeff. of thermal expansion, *ε* (K^−1^)	0.00021	0.00071
Prandtl number, *Pr*	7.08	629
Coeff. of thermal expansion, *ε*_0_ (K^−1^)	0.00021	0.00071

The attenuation for water is taken to be *α* = 0.025(*f*/MHz)^2^ and for sunflower oil *α* = 1.15(*f*/MHz)^1.77^ at 293.15 K. The bulk viscosity is attributable to molecular relaxation, dictating absorption of sound, and has an important influence on the acoustic energy distribution^25^. The bulk viscosities used in this work for the oil, based on Equation (15), are listed in Table 3 at each frequency. The molecular-level need not be described in the equations and a macroscopic variable, *μ*_*B*_, contains adequate information about the relaxation process.

**Table 3:** The attenuation *α* and bulk viscosity *μ*_*B*_ of oil used in the finite element modelling.

Frequency (MHz)	α (Np/m)	*μ*_*B*_ (mPa.s)
1	1.15	98.32
2	3.92	73.22
3	8.04	60.29
4	13.38	51.82
5	19.86	45.62
6	27.42	40.79
7	36.02	36.86
8	45.62	33.86
9	56.20	30.75
10	67.72	28.29
11	80.16	26.11
12	93.51	24.17
13	107.74	22.41
14	122.84	20.82
15	138.80	19.36
16	155.59	18.01
17	173.22	16.76
18	191.66	15.60
19	210.91	14.52
20	230.95	13.51

### Finite element modelling

Information about the absorption, scattering, and reflection of ultrasonic waves in water can in principle allow one to predict the way that the applied field interacts with particles. At and around the oil/water interfaces there are important physical processes occurring that differ dramatically to that of the continuous phase. Numerical modelling is required in order to better understand these processes.

In this work we use a commercially available finite element package, Comsol Multiphysics (version 5.3), for solving the coupled partial differential equations. The volume of the problem under investigation is subdivided into smaller regions, the finite elements, each described by a set of equations that when combined gives a system that describes the overall scientific problem. In particular, we make use of the Thermoviscous Acoustic package in the frequency domain multiphysics interface. Around a particle the viscothermal effects are important in order to be able to accurately describe the acoustic wave propagation. The simulations make use of a full Navier-Stokes model, that is linearised for small perturbations. This allows non-homogeneous boundary conditions around the particles, giving rise to the capability to model decay fields such as heat and shear waves. It is, however computationally very costly. The simulations performed in this work were carried out using a workstation with two Intel Xeon CPU E5-2630v3, 2.40GHz processors and 256Gb of RAM. The full memory capacity and processing power of the machine were utilised to achieve the simulations reported here. The computational cost of resolving the thermal fields is reduced if the particles are closer together because the very low amplitude turning points in the thermal field at larger distances are not required.

The linearised equations governing the acoustic waves are the Navier-Stokes, continuity, and energy equations (16–18):

16 $$i\omega {\rho }_{0}{\bf{u}}=\nabla \cdot (-p{\bf{I}}+\mu [\nabla {\bf{u}}+{(\nabla {\bf{u}})}^{{\bf{T}}}]-[\frac{2}{3}\mu -{\mu }_{{\bf{B}}}][\nabla \cdot {\bf{u}}]{\bf{I}}),$$

17 $$i\omega \rho +{\rho }_{0}\nabla \cdot {\bf{u}}=0,$$

and

18 $$i\omega {\rho }_{0}{C}_{p}T=\nabla \cdot (\kappa \nabla T)+i\omega p{T}_{0}{\varepsilon }_{0},$$

where *ω* is angular frequency, *ρ*_0_ is background density, *μ* is shear viscosity, *μ*_*B*_ is bulk viscosity, *C*_*p*_ is heat capacity at constant pressure, *κ* is thermal conductivity, and *ε*_0_ is the thermal expansion coefficient. The pressure, velocity, temperature, and fluid density variations that oscillate with the applied acoustical wave (*p*, **u**, *T*, and *ρ*, respectively) are superimposed upon the respective background values (indicated by subscript 0). The models are solved in the frequency domain. An incident planar background acoustic field propagates in the z-direction. Thermoviscous acoustics are solved in water and oil domains, based on the equations (16–18) and density variation *ρ* = *ρ*_0_(*β*_*T*_*p* − *ε*_0_*T*), where *β*_*T*_ is isothermal compressibility.

To allow solution of the oil in water problems using the computational resources available, the whole system of particles and water is quartered and symmetry conditions imposed around the *z*-axis. The model is fully 3*D*. The water domain is of radius 6*λ*_*T*_ and a perfectly matched layer is constructed around the model which is 2*λ*_*T*_ thick. A plane wave of amplitude 0.1 MPa is applied in the *z* direction and the centre of the single, or central particle, is at (0, 0, 6*λ*_*T*_).

**Figure 7:**
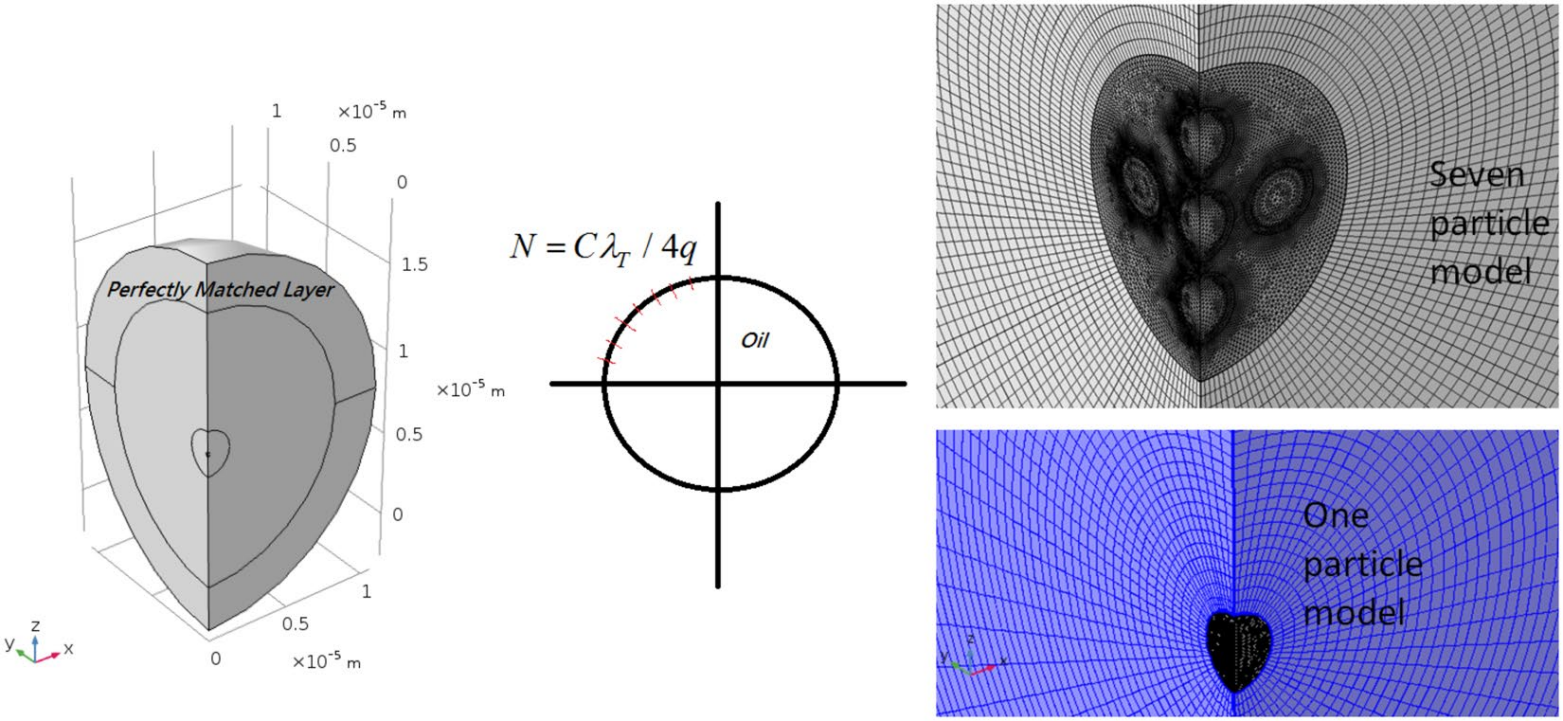
The quarter domain model used in the finite element method. The single particle has a tetrahedral mesh as does the region around a cluster. A swept mesh propagates out radially from the domains belonging to the particles. The distribution in number of elements *N* over a quarter slice of the tetrahedral domain with circumferences *C* allows control over the concentric element sizes through parameter *q*.

The maximum element size is taken as $\ge {\lambda }_{T}\mathrm{/20}$ (see equation (2)) given the analysis of the positions of maxima and minima shown in Fig. 3. We use two different mesh types for single or multiple oil particle in water systems. The single particle has a tetrahedral mesh inside the particle domain and a swept radial mesh propagating outwards from the surface of the particle. A swept mesh is also imposed on the Perfectly Matched Layer. For a cluster of particles, each particle domain has a tetrahedral mesh. A further tetrahedral mesh is created around the cluster with radius 7× particle radius (see Fig. 7, right hand image for the seven particle model). Outside these tetrahedrally meshed areas the water domain exists and has a swept radial meshing. The perfectly matched layer, also with a swept mesh, surrounds the whole region. A mesh is given a maximum growth rate of 1.05, curvation factor of 0.05, resolution of narrow regions 0.8. The tetrahedral domains (only the particle in the single droplet case or the aforementioned area surrounding a cluster distribution) were changed so that at 2*πr*/4 = *C*/4 (where *C* is the circumference of the tetrahedral domain) there are *λ*_*T*_/*x* elements at the boundary. The circumference of the 250 nm diameter particle is nearly exactly the thermal wavelength at 3 MHz. The distribution in number of elements *N* over a quarter slice of the sphere with circumference *C* allows control over the concentric element sizes through parameter *q*. Changing the element maximum size reduces the size of the elements outside the particle (which are controlled by the swept mesh). It was found that the optimal model mesh density produces around 2.7 million degrees of freedom and calibration towards this value is made through changing *q* (typically *q* = 3–6), see Fig. 7. Solutions are found with the built in MUMPS solver that allows the user to avoid any memory allocation issues.

Power dissipation density (power per unit volume) has been calculated using the built-in COMSOL functions for viscous and thermal dissipation. These have been integrated over the specified volumes to obtain the total power dissipation within that volume.
